# Nucleus downscaling in mouse embryos is regulated by cooperative developmental and geometric programs

**DOI:** 10.1038/srep28040

**Published:** 2016-06-20

**Authors:** Elina Tsichlaki, Greg FitzHarris

**Affiliations:** 1 Department of Cell and Developmental Biology. University College London, UK. Gower Street. London WC1E 6BT; 2 Centre Recherche CHUM. Tour Viger. 900 Rue St Denis. Montreal H2X 0B3, Canada; 3 Department Obstérique-Gynécologie. Université de Montréal, Montreal, Canada.

## Abstract

Maintaining appropriate nucleus size is important for cell health, but the mechanisms by which this is achieved are poorly understood. Controlling nucleus size is a particular challenge in early development, where the nucleus must downscale in size with progressive reductive cell divisions. Here we use live and fixed imaging, micromanipulation approaches, and small molecule analyses during preimplantation mouse development to probe the mechanisms by which nucleus size is determined. We find a close correlation between cell and nuclear size at any given developmental stage, and show that experimental cytoplasmic reduction can alter nuclear size, together indicating that cell size helps dictate nuclear proportions. Additionally, however, by creating embryos with over-sized blastomeres we present evidence of a developmental program that drives nuclear downscaling independently of cell size. We show that this developmental program does not correspond with nuclear import rates, but provide evidence that PKC activity may contribute to this mechanism. We propose a model in which nuclear size regulation during early development is a multi-mode process wherein nucleus size is set by cytoplasmic factors, and fine-tuned on a cell-by-cell basis according to cell size.

Establishment and regulation of sub-cellular architecture is a poorly understood aspect of development, and is a particular challenge as cells lessen in size during early embryogenesis. Perhaps the most obvious example of an organelle that must downscale its size during early development is the nucleus, but how this is achieved is unknown. Indeed, the mechanisms that dictate nuclear dimensions are poorly understood in any cellular system^1^,^2^,^3^. Establishing and controlling the correct nucleus size is important for cellular function, having a direct effect on the morphogenesis of organelles, homeostasis, and cell differentiation. Accordingly, alterations in nuclear size and shape are associated with disease states^4^,^5^,^6^,^7^, and maintaining nucleus size is crucial for development^8^,^9^. Thus, regulatory mechanisms must operate that are capable of adapting nucleus size to cell type and then stringently regulating nucleus size to support cellular function during early development.

Recent studies have focused on two distinct but non mutually-exclusive models as to how nuclear size control might be achieved. First, within any given cell type the relative size of the nucleus within the cell (the nuclear/cytoplasmic ratio; N/C) remains constant^8^,^10^,^11^,^12^,^13^,^14^, implying that cytoplasmic volume determines nuclear size. However, direct evidence that cell size impacts nucleus size is scarce. Second, recent work in the Xenopus extract system suggests that changes in nucleus size may be attributable to regulated changes in the cytoplasmic melieu during early development, independent of cell size^15^,^16^. However, the relative contribution of a putative cell-size-sensing mechanism and the influence of developmental stage is difficult to assess in most model systems.

Here we use various strategies, including micromanipulation approaches designed to uncouple cell size from developmental stage, to investigate the relative contribution of cell size and developmental stage in nuclear size control in early embryos. Our data suggest that both mechanisms operate, and we propose a cooperative model in which nuclear size is set by a developmental program, and fine-tuned at any given developmental stage according to cell size.

## Results

### Nuclear scaling during early embryo development

**Figure 1:**
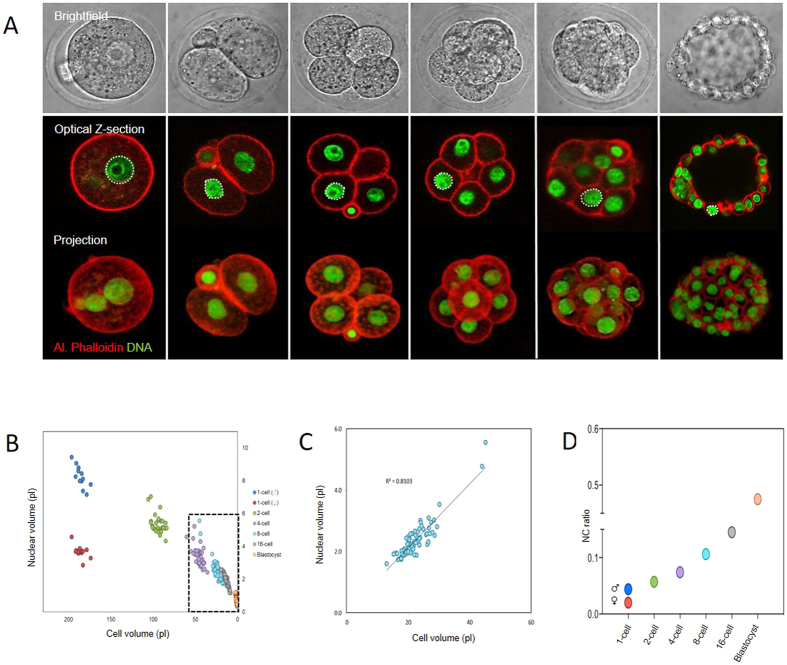
Nuclear volume and N/C ratio during early embryo development. (**A**) Representative images of embryos used to measure volume. (**B**) Quantification of nucleus size, related to cell; size, on a cell by cell basis. Each data point represents an individual cell, colour codes reflect developmental stage. (**C**) ‘Zoomed’ representation of 8-cell embryo data. (**D**) Data expressed as mean nuclear/cytoplasmic ratio. Note that the N/C ratio progressively increases through preimplantation development. 12, 28, 48, 86, 88 and 82 nuclei analysed at 1,2,4,8-cell, morula and blastocyst stage respectively, over the course of 2 replicates.

The mouse preimplantation embryo undergoes a series of reductive cell divisions without intervening cell growth over the course of ~4 days, progressing first to a 16–32 cell morula stage, followed by a 64–128 cell stage blastocyst stage. We first developed methods for accurately calculating nucleus and cell volume during preimplantation development in live and fixed embryos from 3D confocal z-stack images (Fig. 1A, Fig. S1). By examining embryos of each developmental stage we found that nucleus volume decreases ten-fold over the course of preimplantation development, from 8.24 ± 0.19 pL for the male pronucleus in 1-cell stage embryos, to 0.69 ± 0.02 pL for nuclei in blastocysts (Fig. 1B). Therefore, as expected, nucleus size progressively scales down during preimplantation development in mouse.

To begin to understand the mechanisms of nuclear downscaling, we calculated cell volume for each blastomere in embryos during each stage of preimplantation development (Fig. 1, Fig. S1). This allowed us to directly determine the relationship between nucleus size and cell size (N/C ratio) in individual cells within multiple embryos. Analysis of this relationship throughout preimplantation development uncovered two noteworthy features. First, at any given developmental stage there was a tight correlation between nucleus size and cell size, such that N/C ratio was highly consistent between different blastomeres of the same stage (Fig. 1C; Pearsons R^2^ at 8-cell stage = 0.81). This alludes that nuclear size in embryos might depend upon cell size. Second, the value of the N/C ratio increased progressively during development, such that the nucleus occupied a greater proportion of cell volume in later stage embryos (Fig. 1D). This suggests that cell size is not the only determinant of nucleus size, but that there might also be a developmentally-regulated component of nuclear downscaling.

**Figure 2:**
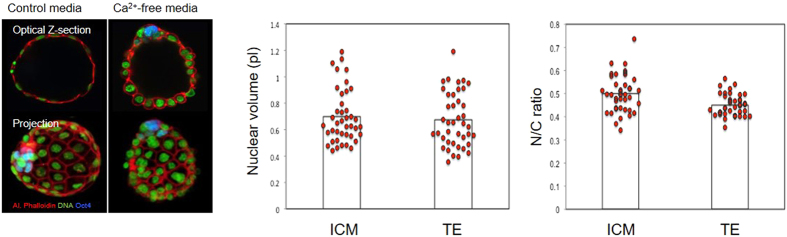
Nuclear downscaling occurs independently of the first cell fate decision. Cells were treated with nominally Ca^2+^-free media for <10 seconds to cause inter-cellular adherence to be partially lost, causing cells to round. Embryos were then fixed and nuclear volume and N/C ratio analysed in Oct4-positive cells, using the same methods as previously. Five cells of each cell type were analysed in 10 blastocysts, across two experimental days.

Blastocyst formation marks the formation of the first two cell lineages; the inner cell mass (ICM) and the trophoectoderm (TE). To determine whether there is any influence of cell lineage upon nucleus size, we labelled blastocysts with OCT4 antibodies, which specifically label ICM cells, to compare N/C ratio in the two cell types (±OCT4 labelling). In preliminary experiments we found it difficult to reliably measure volumes in the flattened outer cells of the blastocyst. We therefore treated embryos with Ca^2+^-free media (~10 secs) prior to embryo fixation to loosen cell adherence and thus cause cell rounding, and ICM cells were identified with OCT4 antibodies. Comparison of ICM and TE revealed no difference in nuclear size between ICM and TE cells (Fig. 2).

Thus in summary, our analysis of N/C ratio across early development alludes to roles for cell size and developmental stage in nucleus size downscaling, in a manner independent upon cell lineage up to blastocyst stage (~64–128 cells).

### Evidence that cell size influences nuclear volume in embryos

We next set out to design experiments to directly test the relative influence of cell size and developmental stage upon nucleus size during early development.

**Figure 3:**
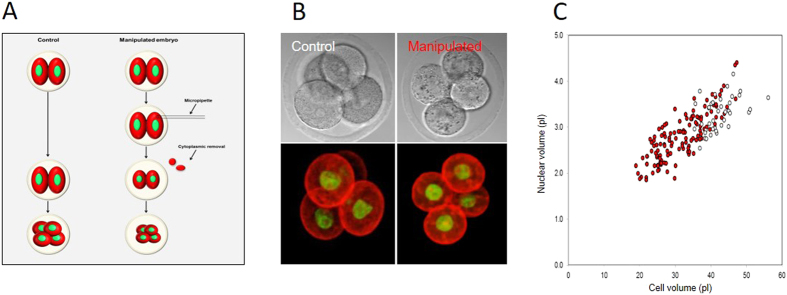
Experimental cytoplasmic reduction to probe the role of cell size in nucleus-size setting. (**A**) Experimental design. (**B**) Examples of control and cytoplasm-reduced embryos fixed and labelled at 4-cell stage. (**C**) Analysis of nuclear and cell volume in the resulting 4-cell embryos. Each point represents one cell. Red manipulated, white controls. Note the tight relationship between cell and nuclear size. R^2^ = 0.67. Data from 82 nuclei over 4 replicates.

First, to determine directly whether cell size influences nuclear volume we used micromanipulation to experimentally reduce cytoplasmic volume. Removal of 20–40% of cell volume using a micropipette from interphase-stage blastomeres had no effect upon nuclear size in the short term (Fig. S2; *P* = 0.61). We wondered however whether reduced cell size might affect the size of a newly forming nucleus at the end of mitosis. Therefore, cytoplasm-reduced 2-cell embryos were allowed to divide, and nucleus size was measured in the resulting 4-cell stage embryos. Cytoplasmic reduction significantly reduced the size of the nucleus in the resulting 4-cell embryos from 3.29 ± 0.06 pL to 2.52 ± 0.04 pL, (*P* = 2.5 × 10^−19^). To examine the sensitivity of this effect we created 4-cell stage embryos of various sizes. Strikingly, nucleus size was tightly correlated with the amount of cytoplasm removed, the nucleus size decreasing correspondingly with cell size over a 2–3 fold change in cell size (Fig. 3). Therefore, reduction of cytoplasmic volume in interphase leads to smaller nuclei following nuclear envelope reformation (NER) at the subsequent mitosis. We note that the relationship between nucleus and cell size is not strictly proportional, since a straight line of best-fit would not cross the origin, suggesting a small cell-size-independent component of nucleus size control. Nonetheless, taken together with the close correlation observed between nucleus and cell size at any given developmental stage (Fig. 1B), we conclude that cell size exerts a major influence upon nuclear size establishment in preimplantation mouse embryos, ensuring that within any given developmental stage the larger cells tend to have larger nuclei.

### Evidence that N/C ratio is developmentally regulated in mouse embryos

**Figure 4:**
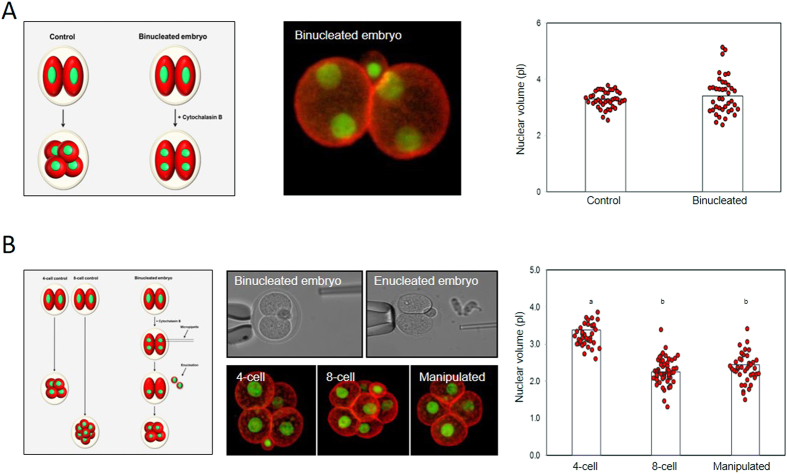
Evidence that N/C ratio is developmentally regulated in mouse. (**A**) Analysis of nucleus size in binucleated embryos. Experimental plan is shown to the left, and an example of a binucleated embryo in the centre panel. Data to the right compares nucleus size in binucleated embryos with normal 4-cell controls. 40 control and binucleated embryos each were examined over 2 days. (**B**) Analysis of nucleus size in ‘double-sized’ blastomeres. Note that the resulting 8-cell-stage nuclei in ’double sized’ blastomeres (n = 42) have nuclei the same size as control 8-cell embryos (n = 56), which are substantially smaller than control 4-cell embryos (n = 40). Data collected over 3 replicates. Different letters indicate P < 0.01 ANOVA (actual values stated in the main text).

Next we set out to determine whether some aspect of developmental stage aside from cell size might contribute to nucleus downscaling. We reasoned that if there were a developmental component to nuclear size control, then nuclear downscaling should occur even in the absence of a cell size decrease. To test this, we performed two series of experiments. First, we generated binucleated embryos in which pairs of 4-cell stage nuclei were encapsulated within cells of 2-cell size (Fig. 4A). Nuclei in binucleated cells had a volume of 3.43 ± 0.10 pL, similar to that from normal 4-cell stage embryos (3.27 ± 0.05 pL, *P* = 0.17), suggesting nuclear downscaling occurs even though there was no reduction in cell size. A possible limitation of this experiment is that the impact of neighboring nuclei is unclear^12^,^14^. Therefore, as a second approach, we produced binucleated 2-cell embryos as previously, and removed one nucleus from each cell, thereby creating embryos in which individual nuclei resided in blastomeres twice the appropriate size. These embryos were allowed to divide, producing blastomeres possessing 8-cell-stage nuclei within ‘4-cell-sized’ cells (Fig. 4B). Nuclei in double-sized blastomeres were the same size (2.36 ± 0.06 pL) as similarly-staged non-manipulated 8-cell stage counterparts (2.24 ± 0.05 pL, *P* = 0.17). Thus, the nucleus scales down according to developmental stage even in the absence of the cell-size reduction that normally happens during development.

In summary our experiments describe two experimentally distinguishable influences upon nucleus size in embryos. First, a cell-size sensing mechanism that works within any given developmental stage to ensure that larger cells have larger nuclei. Second, a developmental program that reduces nucleus size at subsequent mitoses, and is capable of doing so even if the normal cell-size reduction is prevented.

### Nuclear import rates are similar in 2-cell, 4-cell, and 8-cell stage embryos

**Figure 5:**
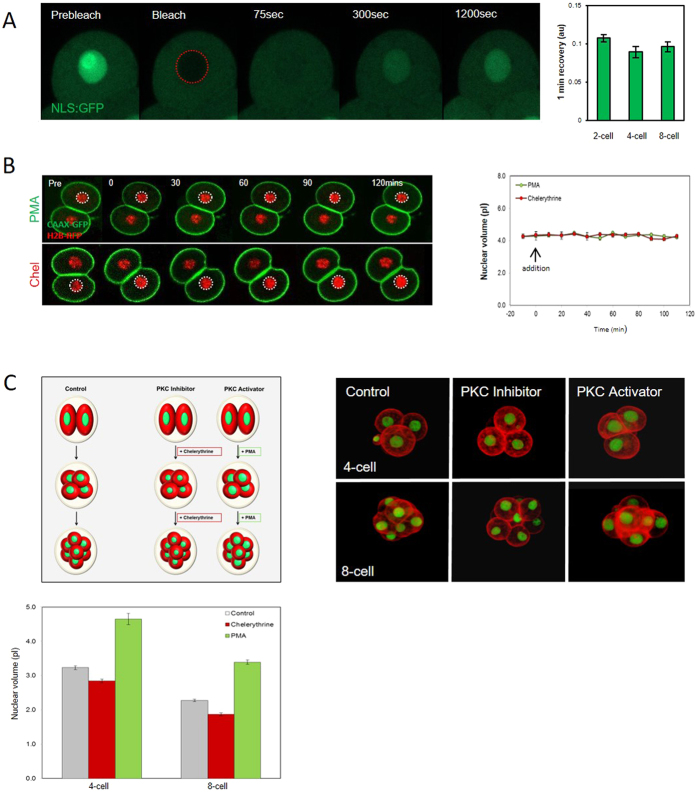
Evidence that PKC, but not nuclear import, contributes to nucleus size-setting. (**A**) NLS::GFP FRAP in 2-cell, 4-cell, and 8-cell stage embryos (n = 10, 9, 9 respectively from 3 replicates). Note there is no difference in the rate of recovery between developmental stages. (**B**) Live imaging of CAAX::GFP and H2B::RFP expressing embryos after interphase addition of PMA or chelyrethrine. (Chel, n = 20, PMA, n = 14 from 2 replicates) (**C**) Analysis of the effect of PMA or chel upon nucleus size following NER. A cartoon is shown of the experimental design, and images show typical examples of each treatment. Note that the treatments cause reciprocal changes in nucleus size. A minimum 37 measurements per experimental group from three replicates. All groups are significantly different to each other within a given developmental stage (P < 0.01 ANOVA for each comparison, actual values stated in main text).

We next wanted to further understand the developmental component of nuclear downscaling. In Xenopus extracts nuclear import rate has recently been linked to nuclear downscaling. Specifically, decreasing import rates during the early divisions in Xenoups correlate with progressively decreasing nucleus size^15^. To examine the importance of nuclear import in nuclear scaling in embryos we first microinjected two-cell-stage embryos with wheat germ agglutinin (WGA) which inhibits nuclear transport^17^. As expected, microinjection of WGA (0.05 mg/ml estimated final concentration) dramatically reduced the size of the nucleus following the subsequent mitosis (Fig. S3). Although WGA can have off target effects, the concentration used had no other impacts upon development to 4 cell stage apart from nucleus size. Thus this experiment confirms the expected result that import participates in establishing nuclear geometry (Fig. S3). To determine whether there are changes in nuclear import rate during early development that might contribute to nuclear downscaling, we performed Fluorescence Recovery After Photobleaching (FRAP) of a GFP-tagged nuclear localization sequence (NLS:GFP)^18^. We found no difference in fluorescence recovery rates between 2-cell, 4-cell and 8-cell embryos (mean recovery in the first minute; 0.107, 0.089 and 0.096 a.u. respectively; P = 0.80; Fig. 5A, Fig. S4). Thus, in notable contrast to the Xenopus extract system, we found no clear evidence of a correlation between nuclear import rate and nucleus size in early mouse embryos.

### PKC inhibitors and activators reciprocally affect nucleus size

Recent work in Xenopus embryo extracts implicated protein kinase C (PKC) activity in nuclear size regulation. Specifically, it was found that pharmacological inhibition or hyperactivation of PKC led to reciprocal effects upon nucleus size^16^. In mouse, intriguingly, a previous detailed analysis found that the abundance of several PKC isotypes decreases progressively during preimplantation development^19^. We therefore wondered whether the PKC decrease may drive nucleus downscaling, and reasoned that increasing PKC activity in embryos should increase nuclear size, whereas inhibition should cause smaller nuclei. To test this we treated embryos with a PKC activator (PMA; 1 ng/ml), or an inhibitor selective for conventional PKC (Chelerythrine; Chel, 1 μM). In contrast to Xenopus extracts, where similar treatments acutely impact nuclear size, we saw no change in nucleus size over several hours of imaging in interphase (Fig. 5B). However, PMA and Chel had striking reciprocal effects on nucleus size following mitosis (Fig. 5C). PKC inhibition by Chel caused a significant decrease in nuclear size (2.85 ± 0.05 pL) compared to controls (3.23 ± 0.05 pL, P = 3.14 × 10^−6^), whereas PKC activation by PMA caused a significant increase in nuclear volume (4.65 ± 0.17 pL, *P* = 2.14 × 10^−12^). Similar reciprocal results were obtained with at the 4–8 cell transition (Fig. 5C). Thus treatment to increase PKC activity promotes larger nuclei at NER, whereas PKC inhibition promotes smaller nuclei, suggesting the decreasing PKC levels in embryogenesis may contribute to nuclear downscaling.

## Discussion

How nuclear size is determined remains poorly understood in any system, and poses an interesting mechanistic puzzle during the reductive divisions of early development. Recent studies predominantly in yeast had extended the long-known association between cell size and nucleus size, implying a mechanistic link^8^,^9^,^11^,^12^,^13^,^14^. Studies in the Xenopus Extract system, on the other hand, highlight the possibility of a role of cytoplasmic contribution that was linked to developmental stage^15^,^16^. Whilst these two mechanisms are in no way mutually exclusive, the huge interspecies differences in cell cycle mechanisms in these settings makes relating these studies difficult, and mechanistic examinations of nucleus-size determination in a mammalian system are few. We set out to examine the role of these two putative systems in mouse embryos, a system in which cells halve in size each cell cycle, whilst the embryo can easily be observed and manipulated ex vivo. Using classical micromanipulation techniques to alter cell size, our data show that cell size and developmental stage are two experimentally dissociable factors that together regulate the nucleus during this process. We envisage that the two mechanisms are independent but co-operate; we propose that gross nucleus size is determined by developmentally regulated cytoplasmic factors that drive nucleus downscaling successive mitoses, and is then fine-tuned by a cell-size sensing mechanism that ensures that within any developmental stage larger cells tend to have larger nuclei.

Clues as to the molecular identity of the cell-size sensing mechanism remain few^1^,^2^,^13^. A recent study identified a dynein-dependent accumulation of membranes at microtubule minus ends adjacent to forming nuclei, that serves as a sensor of the peri-nuclear space^12^. It seems unlikely that the same mechanism should operate in early mouse embryos however, since microtubules are apparently randomly oriented^20^. Given that in mouse embryos nuclei rapidly assume a steady-state diameter that remains stable for the majority of interphase, an appealingly simple explanation is that an as-yet undetermined limiting factor, that contributes to nuclear structure, distributes evenly in the cytoplasm in mitosis, presenting the larger blastomere with more substrate^21^.

More clues are available regarding the developmental shift in nucleus size. Whereas we were unable to find evidence of a developmental regulation in nuclear import, our experiments suggest that PKC activity contributes. This notion is supported by reciprocal effects of PKC activators and inhibitors on nuclear size establishment, both at 4 cell and 8 cell stage, which correspond with the decrease in PKCs previously described in mouse embryos^19^. Although it is important to note that there are clearly species differences in this mechanism between Xenopus and mouse, since PKC activity and nucleus size are inversely correlated in Xenopus Extracts^16^, our data suggest a conserved role for PKC in early developmental nucleus size determination. Though reciprocal PKC activator and inhibitor effects are strong evidence of PKCs involvement, future genetic studies are certainly warranted to further detail this involvement, and ascertain which isoforms are most implicated. Moreover, how PKC activity contributes to nuclear size control in embryos remains to be determined. Intriguingly, relative expression of lamin isotypes changes dramatically during early development^22^ (see also Fig. S5). Since lamins are PKC substrates, we speculate that that PKC activities may modulate lamin incorporation, and that dynamic lamin expression levels and phosphorylation states may thus together impact nuclear size during development. Whereas a simple limiting-factor hypothesis is appealing in terms of controlling nucleus size between cells of the same developmental stage (discussed above), the idea that the availability of a nuclear envelope component (such as a lamin isoform) might be responsible for the overall downscaling that takes place during development appears unlikely, since total nuclear surface area per embryo increases dramatically during the reductive divisions of early embryogenesis (Fig. S6). Finally, it is noteworthy that in mouse the 1-cell embryo possesses two nuclei of highly distinct sizes (see Fig. 1). Since it is hard to imagine that cytoplasmic space or availability of a substrate should limit the growth of only one nucleus, a role for the chromatin state in differential nuclear size setting in embryos is also possible^23^,^24^. Whether chromatin modifications contribute to nuclear downscaling remains to be addressed.

Given the fundamental importance and conceptual simplicity of organelle size-setting and homeostasis, it is remarkable how poorly understood these mechanisms remain. Here we have shown that nucleus size regulation during preimplantation development is unlikely a single pathway, but comprises at least two mechanistically distinguishable functionalities. Our experiments provide one of the first indications of how the embryo goes about the task of adjusting cellular architecture in the face of extreme changes in cell size, and underlines the utility of the mouse embryo as a tool for further dissection of organelle-cell size relationships in dividing and differentiating cells.

## Methods

### Embryo handling, microinjection and micromanipulation

Embryos were obtained from MF1 mice, and embryo culture and microinjection performed as previously^25^,^26^. Enucleation and cytoplasmic removal were performed in media containing 10 μg/ml cytochalasin using an enucleation pipette, and piezo unit (PrimeTech) to breach the zona. Experiments approved by UK Home Office and performed under a PPL to GF. All experiments were in accordance with UK home office guidelines.

### Immunofluorescence and fluorescent proteins

Embryos were fixed for 30 mins in 4% Paraformaldehyde, permeabilised for 10 mins in 0.25% Triton X-100 at 25 °C, and blocked in PBS containing 3% BSA for one hour at 37 °C. Labels used: Alexa Fluor-546 Phalloidin (LifeTech; 1:1000), Hoechst 33342 (LifeTech; 1:1000) or DRAQ5 (LifeTech; 1:300). Primary antibodies used: Oct4 (Santa Cruz 1:300), Lamin A (Abcam; ab2630, 1:200) Lamin B1 (Abcam; ab16048, 1:1000) Lamin B2 (Abcam; ab151735, 1:250) Alexa-fluor secondary antibodies (Life Technologies) were used as appropriate. Blastocysts were subjected to nominally Ca^2+^-free media to cause partial cellular rounding to facilitate volume measurements. Plasmids used were: NLS:GFP in pHM840 (from Addgene), CAAX:GFP pcDNA3.1 (from Guillaume Charras), H2B:RFP in pRN4 (from Alex McDougall). Polyadenylated cRNA was manufactured using mMessage mMachine Ultra (Ambion), as previously^25^.

### Imaging and data analysis

Confocal images were obtained using a Zeiss LSM 510. All live imaging experiments were performed in KSOM at 37 °C/5% CO_2_. For FRAP, photobleaching was performed with 100% 488-nm laser for 20 iterations. Nuclear import rate is expressed as nuclear divided by cytoplasmic fluorescence (both background subtracted), normalised to a prebleach value of 1. For measuring volumes, the N/C ratio of each blastomere was calculated by tracing plasmalemma and nuclear outlines in every z-slice at 2 μm z-intervals. Unless otherwise noted all data were confirmed on at least 3 experimental days. Image analysis was performed using Image J/Fiji. Error bars are s.e.m. Where used, ANOVA was performed using Graphpad, and Students T-tests were used using Excel.

## Additional Information

**How to cite this article**: Tsichlaki, E. and FitzHarris, G. Nucleus downscaling in mouse embryos is regulated by cooperative developmental and geometric programs. *Sci. Rep.* **6**, 28040; doi: 10.1038/srep28040 (2016).

## Supplementary Material

Supplementary Information
