# Supplementary material for: Nucleus downscaling in mouse embryos is regulated by cooperative developmental and geometric programs
Source: Sci Rep. 2016 Jun 20;6:28040. doi: 10.1038/srep28040 (PMC4913252; doi:10.1038/srep28040)

NUCLEUS DOWNSCALING IN MOUSE EMBRYOS IS REGULATED BY COOPERATIVE DEVELOPMENTAL AND GEOMETRIC PROGRAMS

SUPPLEMENTAL FIGURES AND FIGURE LEGENDS

Elina Tsichlaki1 and *Greg FitzHarris1,2,3

1. Department of Cell and Developmental Biology. University College London. Gower Street. London WC1E 6BT

2. Centre Recherche CHUM. Tour Viger. 900 Rue St Denis. Montreal H2X 0B3

3. Department Obstérique-Gynécologie. Université de Montréal. Montreal.

***Correspondence to greg.fitzharris@umontreal.ca**

Figure S1. Methods for calculation of cell end nuclear volume. (A) Co-localisation of the plasmalemma probe CAAX-GFP, and Alexa-phalloidin, in 2-cell stage embryos. Chart to the right shows a fluorescence linescan of the two probes. Note that the signals overlap, validating alexa-phalloidin as a marker of the cell periphery in fixed oocytes. (B) Illustration of section-by-section analysis of cell outlines, as a means of calculating cell and nuclear volume. Volumes were calculated as composites of areas at 2μm z intervals. (C) Embryos co-stained with antibodies against LaminB1 and Hoechst to label the DNA. Note that Hoechst-labelled DNA completely fills the nucleus in embryos, justifying the use of DNA labels as markers of nuclear volume.

Fig S2. Removal of cytoplasm from interphase 2-cell stage embryos does not cause a rapid change in nucleus size during the same cell cycle. Cytoplasm was removed from interphase 2-cell stage embryos using a standard enucleation pipette, and embryos fixed for confocal analysis 2 hours later. Cytoplasmic removals were calculated at 20-40% of total cell volume. Note that cytoplasm reduction did not affect nucleus size within the 2 hours of the experiment, during which the cells remained in interphase. 18 control and 14 cytoplasm-removal cells were examined over the course of two experimental days.

Figure S3. Effect of WGA on nucleus size establishment. (A) Experimental plan is shown inthe cartoon. (B) Analysis of nuclear sizes in 4-cell stage embryos following WGA injection at the 2-cell stage. Note that the nucleus is significantly smaller in WGA-injected embryos (Ttest, $P=1.9 \times 10^{-19}$), illustrating the expected result that WGA prevents nuclear expansion after mitosis. At total of 39 (control) and 34 (WGA) cells were examined over the course of three separate experimental replicates. The experiment shown was performed by microinjecting 1mg/ml WGA (pipette concentration; estimated final cytoplasmic concentration 0.05mg/ml), at which concentration progression to 4 cell stage was unperturbed. Pipette concentration of 2mg/ml or greater appreciably prevented development. 0.2mg/ml injections had no effect upon nucleus size.

Figure S4. FRAP for measuring steady-state nuclear import in 2, 4, and 8-cell embryos. All individual fluorescence recovery traces are shown. Data processed as described in Materials and Methods. Note that photobleaching was slightly more efficient in 4-cell and 8-cell stage embryos than in 2-cells, leading to a lower post-bleach nadir and plateau. As displayed in Fig 5A, the mean rate of change of fluorescence after photobleaching is similar across all groups.

Figure S5. Lamin isoform expression in mouse embryos. 2- 4- 8-cell and morula stage embryos were immunolabelled with isoform-specific lamin antibodies. For any given isoform immunolabelling was performed simultaneously on different developmental stages, and imaging performed with identical microscope settings, to allow quantitative comparision of immunofluorescence levels. Analysis was performed of peak nuclear lamin immunofluorescence level, after background subtraction. Note that nuclear Lamin B1 immunolocalisation increases during preimplantation development, whereas Lamin B2 and Lamin A is reduced. For each isoform 10 embryos per developmental stage were examined across 3 experiments.

Figure S6. Analysis of nuclear surface area during preimplantation development. Dataset the same as presented in Figure 1, re-analysed and presented to indicate surface area. Note that Total nuclear surface area per embryo increases substantially during preimplantation development.

Fig S1. Establishing methods for analysing N/C ratio

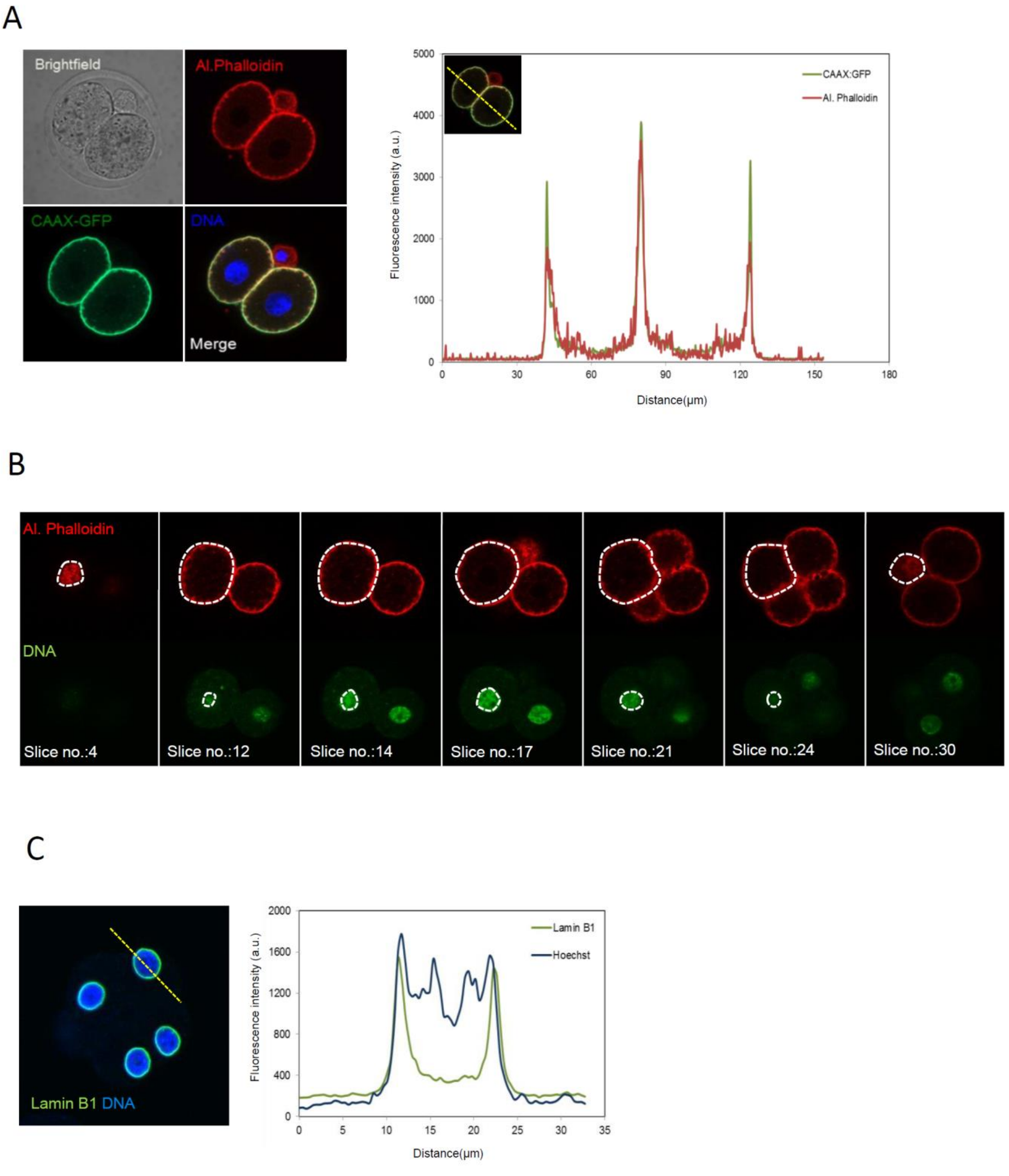

Fig S2. Removal of cytoplasm from interphase 2-cell stage embryos does not lead to a rapid change in nucleus size during the same cell cycle.

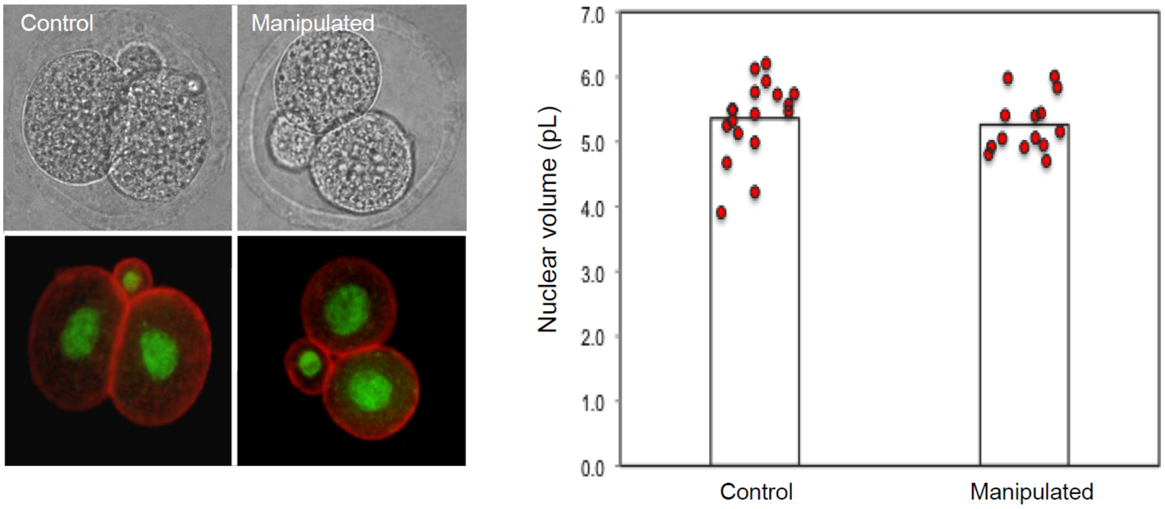

Fig S3. WGA prevents nuclear size establishment

A

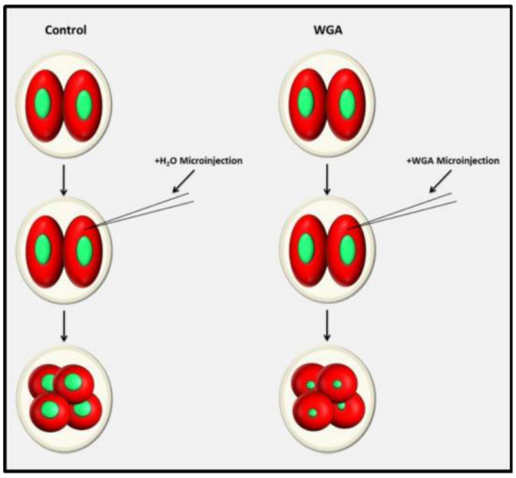

B

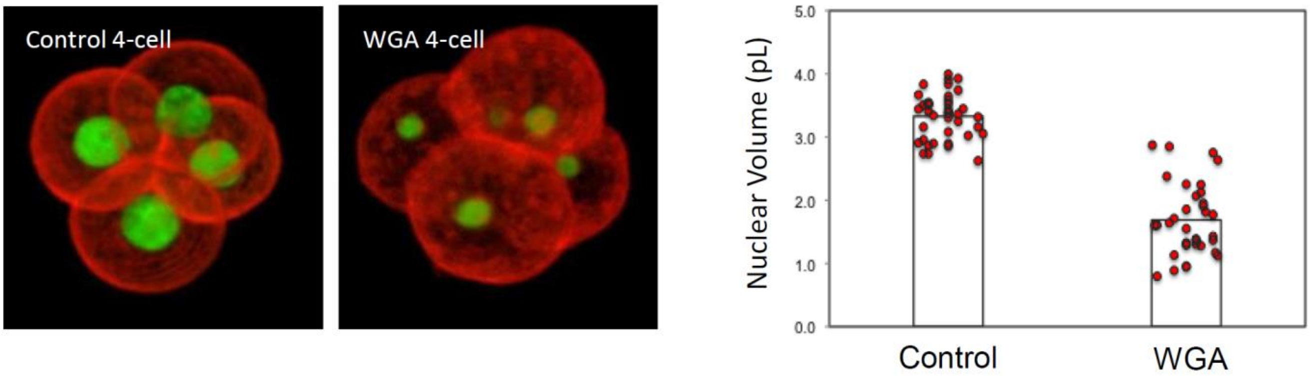

Fig S4. Fluorescence recovery after photobleaching of NLS-GFP in 2,4,8-cell nuclei

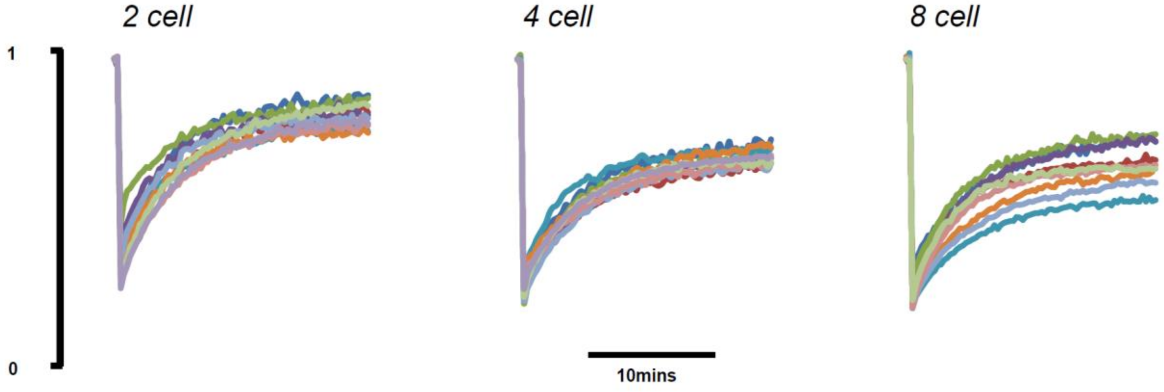

Fig S5. Immunofluorescence analysis of lamin isoform expression during preimplantation development

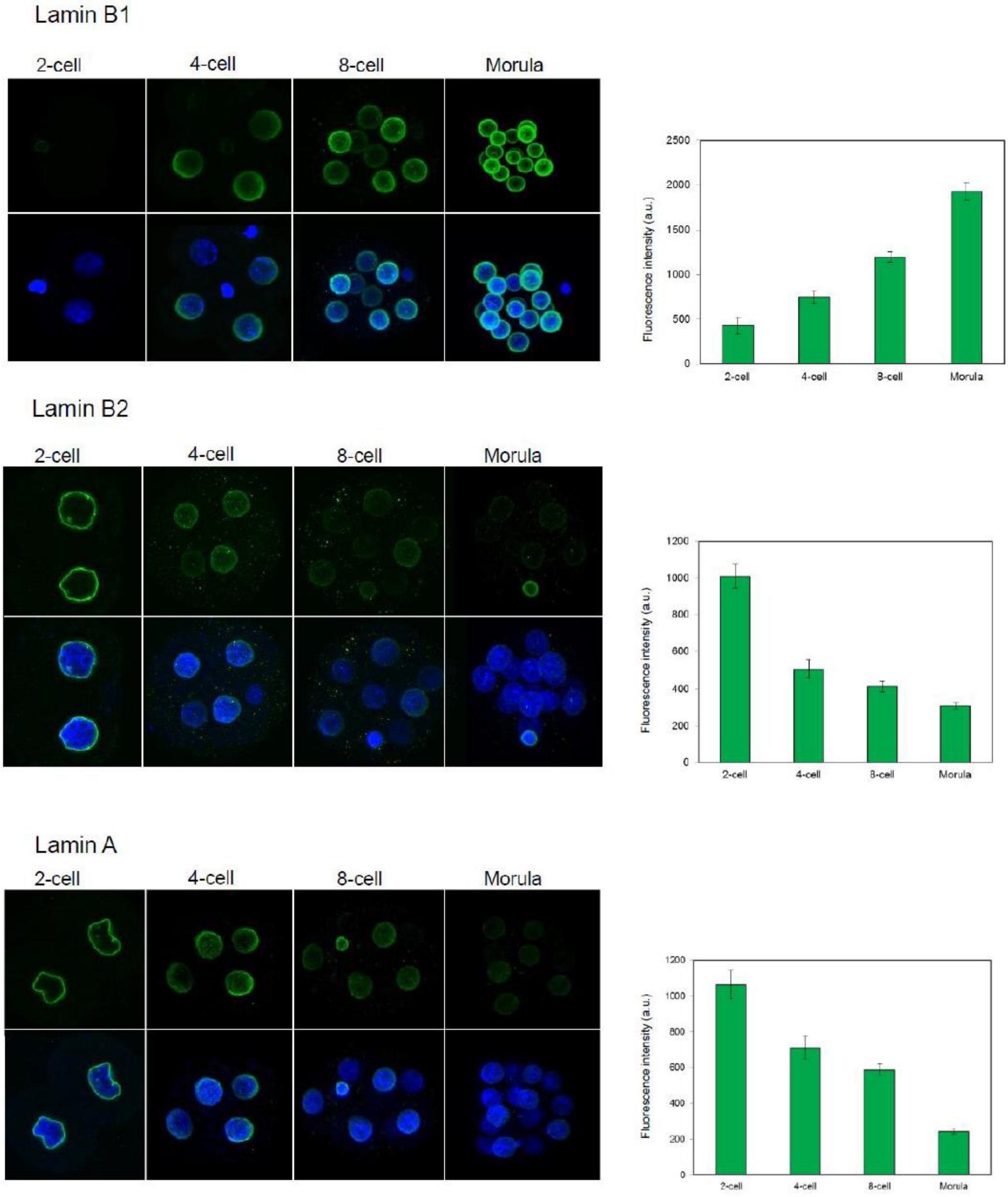

Fig S6. Calculation of total nuclear surface area in early embryos

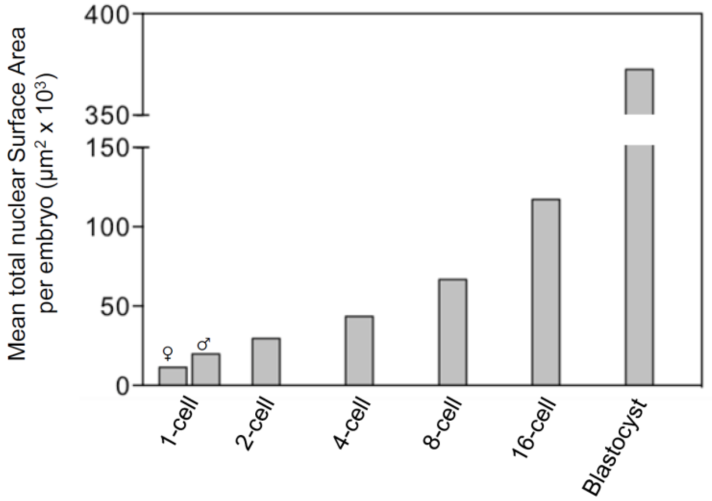

Supplement: Supplementary Information [file srep28040-s1.pdf]
